# Evaluating the Therapeutic Application of Neuromodulation in the Human Swallowing System

**DOI:** 10.1007/s00455-022-10528-z

**Published:** 2022-10-14

**Authors:** Ivy Cheng, Ayodele Sasegbon, Shaheen Hamdy

**Affiliations:** 1 Centre for Gastrointestinal Sciences, Division of Diabetes, Endocrinology and Gastroenterology, School of Medical Sciences, Faculty of Biology, Medicine and Health, University of Manchester, Manchester, UK; 2 Centre for Gastrointestinal Sciences, University of Manchester, Clinical Sciences Building, Salford Royal Foundation Trust, Eccles Old Road, Salford, M6 8HD UK

**Keywords:** Dysphagia, Neuromodulation, Review, Rehabilitation, Stimulation, Treatment

## Abstract

In the last two decades, the focus of neurogenic dysphagia management has moved from passive compensatory strategies to evidence-based rehabilitative approaches. Advances in technology have enabled the development of novel treatment approaches such as neuromodulation techniques, which target the promotion of neurological reorganization for functional recovery of swallowing. Given the rapid pace of development in the field, this review aims to summarize the current findings on the effects of neuromodulation techniques on the human swallowing system and evaluate their therapeutic potential for neurogenic dysphagia. Implications for future clinical research and practical considerations for using neuromodulation in clinical practice will also be discussed.

Swallowing is a complex process mediated by the central nervous system (CNS) involving the brainstem, cerebral cortex, cranial nerves and motoneurons supplying the swallowing musculature [1, 2]. A safe and efficient swallow can only be achieved with the precise coordination of over 50 pairs of muscles, and such coordination is dependent on the integrity of the swallowing neural system. Patients with neurogenic dysphagia suffer from pathology which affects various parts of this system, leading to impaired neurological control of swallowing. Fortunately, the human swallowing motor system appears to be highly plastic, which means that the neural connections are capable of reorganization in response to damages or external neuromodulatory influences. Such reorganization is termed “neuroplasticity”, which plays a key role in the functional recovery of swallowing.

In this review, we will discuss three main neuromodulation techniques that have been proposed as treatments for neurogenic dysphagia, including repetitive transcranial magnetic stimulation (rTMS), transcranial direct current stimulation (tDCS) and pharyngeal electrical stimulation (PES). For each technique, we will briefly introduce the working mechanisms and their neurological and physiological effects on the human swallowing motor system. These will be followed by discussions on clinical studies in patients with post-stroke dysphagia (PSD) and other disease processes. PSD tends to be targeted when studying the effects of novel treatments because it is one of the most common types of neurogenic dysphagia [3] and theoretically involves a single insult, in which neuromodulation techniques can try to overcome via induced neuroplasticity. Other disease processes are rather more complex as they are often degenerative, such that any form of therapy would have to address a variably moving target. Finally, we will discuss some limitations of neuromodulation techniques and the implication for future research and clinical practice, and some considerations for using neuromodulation in clinical practice. This review is developed from a lecture delivered at the 2^nd^ World Dysphagia Summit in 2021. The work presented focuses on the content of the lecture given, which discussed the three main neuromodulation techniques in great detail, and is therefore not intended to be a comprehensive systematic review of all neuromodulation treatments for dysphagia.

## Non-invasive Brain Stimulation (NIBS)

Recent years have seen a growing interest in the therapeutic potential of non-invasive brain stimulation (NIBS) techniques for dysphagia. Repetitive transcranial magnetic stimulation (rTMS) and transcranial direct current stimulation (tDCS) are among the most studied NIBS techniques. The following sections will discuss their mechanisms and effects on the human swallowing motor system in healthy individuals and patients with neurogenic dysphagia.

### Repetitive Transcranial Magnetic Stimulation (rTMS)

Transcranial magnetic stimulation (TMS) is a neurophysiological technique that was initially designed to study the function of the cerebral cortex. In 1985, Baker and colleagues first reported that stimulation of the human motor cortex using TMS resulted in distinct hand and leg movements and such stimulation was relatively painless [4]. This painlessness is an important feature as previous human cortical stimulation studies frequently reported subject discomfort and pain resulted from high voltage electrical currents delivered through surface electrodes [5]. The TMS system comprises an electric pulse generator (stimulator) that is connected to an external coil of wire. The stimulator generates electric currents that flow within the coil, which induces a magnetic field [6]. When this magnetic field is placed near the body, a secondary current that flows in the body tissue will be induced. When this induced current is sufficient in magnitude, it can depolarize the axonal membranes and generate action potentials [7], which then lead to the propagation of signals through nerve conduction within the nervous system [8, 9]. The action potentials evoked by such stimulation can be measured from peripheral muscles as electromyographic (EMG) signals, where they are termed motor evoked potentials (MEPs).

The focality and depth of penetration of TMS-induced electric field are dependent on the geometry of the coil [6]. For example, the currents are induced in circular loops by a circular coil, with the maximum strength occurring under the mean diameter of the coil. By contrast, a figure-of-eight coil, in which two coils are wired such that the stimulator currents flow in opposite directions, induces more focal electric fields than circular coils. The strength of induced fields peaks at the area directly under the centre of the two conjoined coils. Regarding the depth of penetration, in general, the strength of induced field decreases with increasing distance from the coil, and larger coils have greater stimulation depths than smaller ones [6].

Since its first introduction in the early 1980s, TMS has been widely adopted in various areas of human neurophysiology studies. Single-pulse TMS has been used as a diagnostic tool to study and detect abnormalities in the human nervous system [9]. Hamdy et al., conducted the one of the first studies to investigate the cortical representation of the human swallowing system using TMS [10]. They found the mylohyoid, pharynx and oesophagus to be somatotopically represented in the motor and premotor cortices, and such representations are asymmetrical between hemispheres. The hemisphere with larger and more active cortical representation is considered the “dominant” swallowing hemisphere and such dominance is independent of handedness. Hemispheric dominance for swallowing has also been reported, albeit inconsistently, in studies using functional magnetic resonance imaging (fMRI) [11–13]. A possible reason for the less consistent findings from fMRI studies than TMS studies is that such dominance may be task-dependent [14–17]. For example, Mistry et al., found that the right primary motor cortex was strongly activated during water swallowing task, whereas left premotor cortex and supplementary motor cortex were predominantly activated during tongue elevation and saliva swallowing tasks [17]. Their tractography findings on hemispheric dominance correlated with TMS findings for water swallowing task, but not tongue elevation or saliva swallowing tasks. Another reason may be attributed to the different types of data used between TMS and fMRI studies. While TMS studies use individualized data, fMRI studies tend to present averaged data from a group of participants. Given that variations in cortical activation patterns exist across individuals, averaging individual data may lead to a loss in observed dominance patterns among the group.

When TMS is applied repetitively (repetitive TMS; rTMS), long-lasting changes in synaptic efficiency that persist beyond the duration of stimulation can be induced [18]. These changes resemble the strengthening (long-term potentiation) and weakening (long-term depression) of synapses [18, 19], which are thought to be mediated by *N*-methyl-d-aspartate (NMDA) receptors [20, 21]. Although rTMS activates both inhibitory and excitatory cortical circuits, in general, low-frequency (1 Hz or below) rTMS or continuous theta burst TMS suppress cortical excitability, whereas high-frequency (3 Hz or above) rTMS or intermittent theta burst TMS enhance cortical excitability [22, 23]. This ability to modulate synaptic plasticity has made rTMS a potentially viable tool for the neruorehabilitation following neurological damage such as that caused by strokes [24].

Several safety guidelines for rTMS have been published since 1998 [25–27]. The most undesirable adverse effect associated with rTMS is induced seizure due to the spread of excitation across the brain. Fortunately, the occurrence of induced seizures is extremely rare when the safety guidelines are followed (estimated risk less than 0.03%), and no permanent damage has been reported [26]. Other reported adverse effects are relatively mild, including effects on cognition and mood, transient effects on hormones and lymphocytes, transient auditory threshold shift, pain and headache, skin burns when applied over conductive surface electrodes or implants and psychological consequences of induced seizure [25, 26]. Importantly, the latest guideline suggested that rTMS using figure-of-eight coil is considered safe in individuals who are taking drugs that lower seizure threshold, and individuals with cardiac pacemakers, vagus nerve stimulators, and spinal cord stimulators, as long as it is applied at least 10 cm away from electronic components.

#### Healthy Participant Studies

Of relevance to dysphagia rehabilitation is the effects of rTMS on the human swallowing motor cortex. Gow et al., found that high-frequency 5 Hertz (Hz) rTMS increased the excitability of the pharyngeal motor cortex and the effects lasted for more than 60 min [28]. On the other hand, low-frequency 1 Hz rTMS reduces the excitability of the pharyngeal motor cortex and such suppression is associated with disruptions in swallowing behaviour (reduced swallowing reaction time and accuracy) for approximately 60 min [29]. This temporary disruption of the swallowing system was termed a “virtual lesion”, which is a powerful tool for studying treatment effects in healthy individuals before clinical studies because the disruptions resemble those caused by brain damage but are temporary. In a further study, Jefferson et al., demonstrated that 5 Hz rTMS applied over the contralesional hemisphere could reverse the neurological and behavioural disruptions induced by a “virtual lesion” [30]. Taken together, these findings provided empirical evidence that the human swallowing motor cortex is plastic and susceptible to rTMS.

#### Neurogenic Dysphagia (Stroke) Studies

In patients with PSD, several studies have shown that rTMS induces long-lasting changes in the swallowing motor system. These changes include increased cortical representation and excitability [31, 32] and enhanced pharyngeal sensory conduction [33]. Moreover, the effects of rTMS on cortical excitability are specific to stimulation frequency and hemisphere [34]. Du et al., found that 3 Hz ipsilesional rTMS increased mylohyoid cortical excitability in the stimulated hemisphere only, but 1 Hz contralesional rTMS reduced excitability of the stimulated hemisphere and increased cortical excitability in the unstimulated hemisphere [34].

Over the last two decades, a number of randomized controlled trials (RCTs) have been conducted to explore the therapeutic value of rTMS for the treatment of dysphagia (Table 1). Recent systematic reviews and meta-analyses suggested that rTMS may have potential benefits for neurogenic dysphagia [35, 36]. However, the stimulation protocol, in particular the stimulation hemisphere and frequency, remains the centre of debate for rTMS treatments. In general, these protocols are derived based on two models of stroke recovery [37]. The first one is the vicariation (or substrate compensatory strategy) model, which is based on the findings from the study by Hamdy et al. [38]. They found that functional recovery of swallowing following unilateral hemispheric stroke is associated with an increase in cortical representation of the unaffected hemisphere, suggesting that the reorganization of the intact hemisphere is critical to functional recovery.

**Table 1:** Summarized findings on the effects of rTMS for post-stroke dysphagia

Study	Stimulation protocol	Number of sessions	Hemisphere	Target	Sample size	Post-stroke duration (years)	Main findings
*High-frequency contralesional rTMS*							
Park et al. 2013 [39]	**5 Hz** 90% hand RMT 500 pulses	10 days	Contralesional	Pharyngeal MC	Active: 9 Sham: 9	61.9 (21.6)	rTMS reduced prevalence of aspiration and pharyngeal residue
Michou et al. 2014 [40]	**5 Hz** 90% hand RMT 250 pulses	Single session (Cross-over)	Contralesional	Pharyngeal MC	6	212.1 (164.5)	No significant treatment effects on aspiration/penetration or swallowing timings
Cabib et al. 2020 [33]	**5 Hz** 90% hand RMT 250 pulses	Single session (Cross-over)	Contralesional	Pharyngeal SC	12	493.1 (672.4)	No significant treatment effects on aspiration/penetration or swallowing timings
*Low-frequency contralesional rTMS*							
Lim et al. 2014 [44]	**1 Hz** 100% mylohyoid RMT 1200 pulses	10 days	Contralesional	Pharyngeal MC	CDT + rTMS: 14 CDT: 15	32.4 (12.5)	rTMS reduced penetration and aspiration and improved swallowing function for liquid consistency
Tarameshlu et al. 2019 [45]	**1 Hz** 120% mylohyoid RMT 1200 pulses	5 days	Contralesional	Mylohyoid MC	CDT: 6 rTMS: 6 CDT + rTMS: 6	96 (54)	Combined treatments superior to either rTMS or CDT alone
Unluer et al. 2019 [46]	**1 Hz** 90% mylohyoid RMT 1200 pulses	5 days	Contralesional	Mylohyoid MC	CDT: 15 CDT + rTMS: 13	103.8 (45.1)	No significant group differences in treatment effects on aspiration/penetration
*High-frequency ipsilesional rTMS*							
Khedr et al. 2009 [31]	**3 Hz** 120% hand RMT 300 pulses	5 days	Ipsilesional	Esophageal MC	Active: 14 Sham: 12	5–10	rTMS reduced dysphagia severity and the effects lasted for up to 2 months rTMS increased esophageal cortical representation in the damaged hemisphere and cortical excitability in both hemispheres
Cheng et al. 2017 [47]	**5 Hz** 90% tongue RMT 3000 pulses	10 days	Ipsilesional	Tongue MC	Active: 11 Sham: 4	1251 (618)	No significant group differences in biomechanical measures of swallowing
*Comparisons among different frequencies and hemispheres*							
Xie et al. 2022 [48]	**iTBS** 100% suprahyoid RMT 600 pulses **10 Hz rTMS** 100% suprahyoid RMT 1200 pulses	Single session	Ipsilesional	Suprahyoid MC	iTBS: 24 rTMS: 23 No sham	27.5 (9.9)	No significant group differences in improvement of swallowing function
Kim et al. 2011 [49]	**5 Hz** 100% mylohyoid RMT 1000 pulse **1 Hz** 100% mylohyoid RMT 1200 pulses ** + CDT**	10 days	**5 Hz** Ipsilesional **1 Hz** Contralesional	Mylohyoid MC	5 Hz: 10 1 Hz: 10 Sham: 10	29.9 (23.9)	rTMS improved swallowing function 1 Hz rTMS superior to 5 Hz rTMS in reducing aspiration and penetration
Du et al. 2016 [34]	**3 Hz** 90% mylohyoid RMT 1200 pulses **1 Hz** 100% mylohyoid RMT 1200 pulses	5 days	**3 Hz** Ipsilesional **1 Hz** Contralesional	Mylohyoid MC	3 Hz: 13 1 Hz: 13 Sham: 12	12.6 (15.5)	Both 3 Hz and 1 Hz rTMS improved swallowing function and the effects lasted for up to 3 months 1 Hz rTMS reduced cortical excitability of affected hemisphere but increased cortical excitability of unaffected hemisphere 3 Hz rTMS increased cortical excitability of affected hemisphere only
Khedr et al. 2010 [50]	**3 Hz** 130% hand RMT 300 pulse (each hemisphere)	5 days	Both	Esophageal MC	Active: 11 Sham: 11	32.2 (16.8)	rTMS reduced dysphagia severity and the effects lasted for up to 2 months
Park et al. 2017 [51]	**10 Hz** 90% mylohyoid RMT 500 pulses (each hemisphere)	10 days	**Bilateral** Both **Unilateral** Ipsilesional	Mylohyoid MC	Bilateral: 11 Unilateral: 11 Sham: 11	35 (33.6)	Bilateral rTMS superior to unilateral rTMS and sham rTMS in improving swallowing function
Zhang et al. 2019 [32]	**10 Hz** 110% mylohyoid RMT 900 pulses **1 Hz** 80% mylohyoid RMT 900 pulses ** + NMES**	10 days	**Bilateral** 10 Hz ipsilesional, then 1 Hz contralesional **10 Hz** Ipsilesional **1 Hz** Contralesional	Mylohyoid MC	Bilateral: 16 10 Hz: 16 1 Hz: 16 Sham 10 Hz: 16	23.7 (7.3)	Bilateral rTMS superior to unilateral rTMS in improving swallowing function and cortical excitability

*Data are presented in Mean (SD)

Significance was set at 0.05

*CDT* conventional dysphagia therapy, *RMT* resting motor threshold, *MC* motor cortex, *SC* sensory cortex, *NMES* neuromuscular electrical stimulation

Based on the vicariation model, high-frequency rTMS was applied to the unaffected hemisphere to promote reorganization in three RCTs [39–41]. Park et al., found that 5 Hz rTMS applied over contralesional pharyngeal motor cortex reduced prevalence of aspiration and pharyngeal residue in subacute (between 1 and 3 months) stroke patients [39]. However, two other randomized controlled cross-over studies did not find significant treatment effects on swallowing safety or efficiency in chronic (> 3 months post event) stroke patients [33, 40]. These contradictory findings may be attributed to the differences in patient characteristics (subacute versus chronic stroke), stimulation duration (10 days versus single sessions) and study designs (parallel groups versus cross-over).

Another model of recovery is the interhemispheric inhibition model, which is based on findings that when one hemisphere is damaged, the intact hemisphere would exert excessive inhibition over the damaged one potentially hindering its recovery [42, 43]. Therefore, several rTMS protocols have been developed with the aims of resolving any interhemispheric imbalance. Some studies applied low-frequency 1 Hz rTMS over the contralesional hemisphere [44–46], but mixed findings have been reported. Two studies found that 1 Hz rTMS combined with conventional dysphagia therapy reduced dysphagia severity and risks of penetration and aspiration [44, 45]. However, Unluer et al., did not find such superiority with combined treatments [46]. Other studies applied high-frequency (3 Hz or 5 Hz or 10 Hz) rTMS over the ipsilesional hemisphere [31, 47]. Khedr et al. [31] reported that 3 Hz rTMS reduced dysphagia severity in acute stroke patients, but the study by Cheng et al. [47] did not find any treatment effects of rTMS on swallowing biomechanics in chronic stroke patients. These mixed findings may be attributed to the heterogeneity in treatment protocols and sample population.

When comparing the effects of different rTMS protocols, Xie et al., found that both iTBS and 10 Hz rTMS improved swallowing function in stroke patients [48]. Of note, this study did not include a sham comparison group such that spontaneous recovery cannot be ruled out. Du et al., did not find significant differences between the treatment effects of high-frequency ipsilesional rTMS and low-frequency contralesional rTMS [34]. However, Kim et al., found that high-frequency ipsilesional rTMS was more beneficial than low-frequency contralesional rTMS in reducing the risks of aspiration and penetration [49]. Finally, several studies suggested that bilateral rTMS is more effective than unilateral rTMS in improving swallowing function, reducing penetration and aspiration, and reducing dysphagia severity [32, 50, 51].

Several systematic reviews and meta-analyses have attempted to synthesise an overall treatment effect of rTMS based on evidence from published clinical trials [35, 52–56]. The findings from these studies suggested that rTMS has moderate to large treatment effects in improving swallowing in patients with PSD. Regarding the stimulation frequency and hemisphere, a recent meta-analysis found that the effects of rTMS analysed based on these two parameters were not significant [56]. However, given that rTMS protocols were derived based on different recovery models, analysing the effects of frequency and hemisphere as separate entities may lead to over-generalization. Therefore, Cheng et al., evaluated the effects based on both entities in the same subgroup analysis and found that high-frequency ipsilesional rTMS showed large treatment effects, whereas contralesional low-frequency rTMS and bilateral rTMS showed moderate treatment effects [35]. Nonetheless, the number of clinical trials that were available for these reviews and meta-analyses were limited and the heterogeneity among studies made it difficult to draw definitive conclusions regarding clinical efficacy.

#### Neurogenic Dysphagia–Other Disease Processes

In patients with Parkinson’s disease (PD), Khedr et al., found that 20 Hz rTMS applied over the hand motor cortex of both hemispheres for 10 days improved the timing of hyoid bone elevation and pharyngeal transit time for fluid swallowing [57]. Moreover, a recent pilot study compared the effects of different neuromodulation treatments for PD patients [58]. In this RCT study with cross-over design, twelve PD patients were randomized to receive either 1 Hz rTMS, 5 Hz rTMS or PES. The results suggested potential benefits of these treatments and that they were well tolerated by all patients. Finally, apart from stroke and PD patients, Park et al., found that rTMS can improve swallowing function and increased cortical activation during swallowing in elderly patients with dysphagia [59]. These findings suggested that rTMS may also benefit patients with other neurogenic dysphagia.

### Cerebellar rTMS

Apart from cortical stimulation, some studies have explored the effects of cerebellar stimulation. This is due the cerebellum being known to be involved in modulating swallowing [60]. Jayesakeran et al., found that the cerebellum exerts modulatory effects on the pharyngeal motor cortex, which suggested that stimulation of the cerebellum may result in modulation of the swallowing motor system, making cerebellar stimulation a potential tool for dysphagia rehabilitation [61]. Further studies have identified that 10 Hz rTMS is optimal for inducing excitatory effects on the pharyngeal motor cortex [62] and reversing the neurological and behavioural disruptions induced by “virtual lesion” to the pharyngeal motor cortex [63]. Moreover, Sasegbon et al., conducted a series of studies and found that bilateral cerebellar rTMS was more effective in provoking pharyngeal cortical excitation and reversing the impacts of “virtual lesion” than unilateral cerebellar rTMS [64]. Interestingly, stimulation of the midline of cerebellum exerts a suppressive effect on pharyngeal cortical activity and swallowing behaviour [65]. In a case-controlled study, a 67-year-old patient with right posterior fossa infarction received both active and sham cerebellar rTMS [66]. The results showed that cerebellar rTMS reduced penetration and aspiration and increased pharyngeal cortical excitability, which makes it a potential treatment for dysphagia. A recent pseudo-randomized controlled trial study with 147 stroke patients reported improved swallowing function and reduced severity of dysphagia following 5 Hz ipsilesional rTMS, 5 Hz contralesional rTMS and 5 Hz cerebellar rTMS [67]. However, due to some methodological issues with this study in which the control group was not included in the randomization process, the conclusions on the treatment effects remained controversial. Further RCTs are needed to understand the clinical efficacy of cerebellar rTMS. Finding from an ongoing double-blinded RCT which aims at identifying the feasibility of using cerebellar rTMS as a treatment for dysphagia in acute or subacute stroke patients and the optimal treatment regime will provide valuable insights into the therapeutic value of cerebellar rTMS for PSD [68].

## Transcranial Direct Current Stimulation (tDCS)

Transcranial direct current stimulation (tDCS) is a form of transcranial electrical stimulation (tES) which delivers electric current onto the brain through surface electrodes, which involve one or more positive (anode) and negative (cathode) electrodes. During tDCS, the “active” electrode is placed over the target area while the “return” electrode is placed over another cranial region or body part. Unlike rTMS which directly depolarizes neurons, tDCS produces internal electric fields that are lower than the intensity required to evoke action potentials in a resting cell [69]. It changes the threshold for discharge of stimulated neurons and modulates the firing rate of individual neurons with relatively weak current [70, 71]. When applied for a sufficient duration, tDCS can induce changes in cortical excitability that last longer than the stimulation duration [72]. The underlying mechanisms for such long-lasting changes remain largely unknown, but pharmacological studies have suggested that these effects may be mediated by *N*-methyl-*D*-aspartic acid (NMDA) receptors [73]. A traditional albeit simplistic view of the brain effects of tDCS suggests that the anode increases the excitability of the cortical region directly beneath it whereas the cathode decreases excitability. As this occurs, the areas between electrodes remain relatively unaffected. However, recent studies have diverged from this view and found that the neuromodulatory effects of tDCS are dependent on several factors, including the size and position (montage) of electrodes and distance between electrodes [74, 75]. Specifically, Moliadze et al., found that increasing inter-electrode distance reduces the neuromodulatory effects of tDCS [74]. Moreover, the position and size of the “return” electrode influence the electric field distribution of the entire cortex [75]. Therefore, both “active” and “return” electrodes may have neuromodulatory effects on the cortex and they should not be considered separately.

There are two other forms of tES, namely transcranial alternating current stimulation (tACS) and transcranial random noise stimulation (tRNS). Both tACS and tRNS deliver low intensity alternating current over the brain to manipulate the cerebral cortex [76]. They can manipulate the cortical neural oscillations by entraining such oscillations to a specific frequency (or a range of frequencies for tRNS) [77]. A pilot study has reported preliminary evidence that gamma (70 Hz) tACS and full-spectrum tRNS were effective in enhancing pharyngeal cortical excitability in healthy individuals [78].

Given that these three forms of tES use weak current to modulate neural activity, they are considered relatively safe compared to rTMS. A safety guideline for tES have been published in 2017 which suggested that low intensity tES (< 4 mA; < 10,000 Hz; electrode size < 100 cm^2^; duration < 60 min a day) is a safe technique [79]. No serious adverse effects have been reported among more than 10,000 patients who received active tDCS [80]. Other reported adverse effects include itchiness at the scalp, burning sensation, headache, tingling sensation, sleepiness, difficulties of concentration, mild fatigue, skin redness, and dizziness [81]. These adverse effects were transient and typically resolved once tDCS stopped.

### Healthy Participant Studies

Jefferson et al., conducted the first study to investigate the effects of tDCS on the human swallowing motor system [82]. They found that 10 min of 1.5 mA and 20 min of 1 mA anodal tDCS enhanced whereas 10 min of 1.5 mA cathodal tDCS suppressed the excitability of pharyngeal motor cortex. Following this, a number of studies on healthy volunteers have shown that anodal tDCS applied over the pharyngeal motor cortex induces changes in the human swallowing motor system, including increased cortical excitability [83, 84], increased cortical activation in both hemispheres [85], improved swallowing behaviour [85, 86] and changes in swallowing pressure [84]. Finally, tDCS has been shown able to reverse the neurological and behavioural disruptions caused by rTMS-induced “virtual lesion” to the pharyngeal [87] and mylohyoid motor cortex [88]. These findings suggested that tDCS can alter the human swallowing motor system, making it a potentially viable neuromodulatory tool for dysphagia rehabilitation.

### Neurogenic Dysphagia Studies

#### Stroke Studies

A number of RCTs have examined the therapeutic value of tDCS for dysphagia rehabilitation [89–97]. These RCTs studied anodal tDCS as an adjunctive therapy to conventional swallowing therapy instead of a standalone treatment. The stimulation durations ranged from 20 to 30 min for 4 to 48 days. Similar to rTMS, both unilateral (ipsilesional and contralesional) and bilateral stimulation have been studied. Table 2 summarizes the findings reported in published RCTs on the effects of tDCS for PSD.

**Table 2:** Summarized findings on the effects of tDCS for post-stroke dysphagia

Study	Stimulation protocol	Number of sessions	Hemisphere (C/I)	Stimulation target	Sample Size	Post-stroke duration (days)*	Main findings
Kumar et al. 2011 [89]	2 mA/30 min + CDT	5 days	Contralesional	Sensorimotor cortex	Active: 7 Sham: 7	(1.8)	tDCS reduced dysphagia severity
Yang et al. 2012 [90]	1 mA/20 min + CDT	10 days	Ipsilesional	Pharyngeal MC	Active: 9 Sham: 7	(10.2)	tDCS reduced dysphagia severity
Shigematsu et al. 2013 [91]	1 mA/20 min + CDT	10 days	Ipsilesional	Pharyngeal MC	Active: 10 Sham: 10	(58.8)	tDCS reduced dysphagia severity
Ahn et al. 2017 [92]	1 mA/20 min + CDT	10 days	Both	Pharyngeal MC	Active: 13 Sham: 13	35741)	No significant group differences in treatment effects
Suntrup-Krueger et al. 2018 [93]	1 mA/20 min + CDT	4 days	Contralesional	Pharyngeal MC	Active: 29 Sham: 30	(0.3)	tDCS reduced dysphagia severity
Pingue et al. 2018 [94]	2 mA/30 min + CDT	10 days	**Anodal** Ipsilesional **Cathodal** Contralesional	Pharyngeal MC	Active: 20 Sham: 20	< 30	No significant group differences in treatment effects
Wang et al. 2020 [95]	1 mA/20 min + Balloon dilation + CDT	20 days	Both	Esophageal MC	Active: 14 Sham: 14	(42.5)	tDCS reduces dysphagia severity and pharyngoesophageal segment opening
Sawan et al. 2020 [96]	2 mA/30 min + CDT	10 days	**Active group 1** Contralesional **Active group 2** Dominant for 5 days, then non-dominant for 5 days	Pharyngeal MC	Active: 20 Sham: 20	(5.0)	tDCS reduced dysphagia severity
Mao et al. 2021 [97]	1.6 mA/20 min + CDT	48 days	Contralesional	SMC	Active: 20 Sham: 20	103.5 (69.9)	tDCS reduced dysphagia severity

*Data are presented in Mean (SD)

Significance was set at 0.05

*CDT* conventional dysphagia therapy, *MC* motor cortex, *SMC* sensorimotor cortex

The study by Kumar et al., was one of the first to evaluate the effects of unilateral anodal tDCS in patients with PSD [89]. Their results showed that anodal tDCS (2 mA for 30 min) applied over the contralesional sensorimotor cortex reduced the functional severity of dysphagia, as reflected in reduced Dysphagia Outcome and Severity Scale (DOSS) score, and patients tolerated the procedures well without adverse effects. Since then, other RCTs have also reported positive changes in swallowing function [90, 91, 93, 97] and nutritional status [97] following anodal tDCS. Notably, Suntrup-Krueger et al., reported that the cortical activation in the stimulated hemisphere increased following tDCS [93]. This finding provided evidence that tDCS can induce long-term plasticity effects in the cortical networks for swallowing in patients with brain damage.

Although unilateral anodal tDCS have shown preliminary benefits for PSD regardless of stimulation sites and intensity, mixed findings have been reported on the effects of bilateral tDCS protocols. Ahn et al., found that the outcomes of anodal tDCS applied over both hemispheres were not superior to sham stimulation [92]. Similarly, a study by Pingue et al., showed that while ipsilesional anodal tDCS and contralesional cathodal tDCS led to subtle improvements in swallowing, the differences between active and sham groups were not statistically significant [94]. By contrast, Wang et al., reported positive findings in brainstem stroke patients with cricopharyngeal muscle dysfunction [95]. They found that bilateral anodal tDCS, when combined with balloon dilation therapy and conventional swallowing therapy, was superior to sham tDCS in improving swallowing function and pharyngoesophageal segment opening.

Given the growing interest in tDCS and the mixed findings in this area, several systematic reviews and meta-analyses have evaluated its clinical efficacy for PSD in the recent 2 years [35, 36, 98–100]. Collectively, these reviews showed that tDCS has modest but promising beneficial effects for this population.

#### Other Disease Processes

Apart from stroke patients, some studies have proposed that tDCS may be beneficial to patients with multiple sclerosis (MS). A case report study by Cosentino et al., suggested that anodal tDCS (2 mA for 25 min) applied over the pharyngeal motor cortex have some clinical benefits for MS patients and the improvement persisted for up to 1 month post-stimulation [86]. In a further RCT study, Restivo et al., found that the same anodal tDCS protocol applied over the dominant (stronger) pharyngeal motor cortex improved swallowing function in MS patients with severe dysphagia [101].

## Pharyngeal Electrical Stimulation (PES)

Pharyngeal electrical stimulation (PES) is a peripherally applied neuromodulation technique that aims to encourage beneficial neuroplastic changes in the sensory and motor cortices by electrically stimulating the pharyngeal mucosa [102]. Electrical stimulation is delivered through a catheter containing electrodes. The catheter is inserted either trans nasally or trans orally and positioned such that its electrodes sit in the mid pharynx and make good contact with the surrounding mucosa [103]. Once adequately positioned electrical energy is provided by an electrical generator at a frequency of 5 Hz [104]. The threshold for the amount of electrical energy required is determined by instructing individuals to indicate when they first feel the induced sensation from the catheter and when that sensation becomes uncomfortable. This is repeated three times before the individualized therapeutic setting is chosen as 75% of the average maximum tolerated threshold [104]. PES is a safe technique. Despite its use in several healthy participants and neurogenic dysphagia studies, no seizures or other serious device-related adverse events have been reported [105, 106]. However, these studies have pre-emptively attempted to mitigate the potential occurrence of adverse events by excluding study participants with implanted defibrillators, severe respiratory compromise or participants on oxygen therapy.

Administering electrical energy to the pharynx modulates the activity of both the CNS and peripheral nervous system (PNS). With respect to the CNS, there is an increase in sensory (and motor) input into the nucleus tractus solitarius (NTS) within the medulla oblongata, which along with the efferent nucleus ambiguous (NA), constitutes the central pattern generator (CPG) [107]. Impulses then travel bilaterally to the sensory cortices modulating sensory activity within the cerebral higher centres both in health and in individuals with neurogenic dysphagia [102]. It is thought that this sensory modulation leads to (sensori-)motor cortical modulation. Studies have shown increases in activity within the pharyngeal motor cortices following PES [103]. However, the precise neurological pathways through which the sensory and motor cortices communicate are unclear. Potential pathways include intracortical communication between sensory and motor areas [108] or communication between these two areas via the brainstem [109, 110]. Regarding the PNS, it has been shown that PES increases salivary levels of the neurotransmitter substance P [111]. In one of its roles, substance P is thought to enhance the swallow reflex [111, 112]. Studies have shown an association between low levels of substance P and PSD [112]. This finding indicates a potential additional peripheral neuromodulatory mechanism of action for PES whereby following stimulation there is peripheral modulation and enhancement of sensory input into the brainstem.

Drinking carbonated water is another method that has been shown to increase pharyngeal sensory inflow into the swallowing sensorimotor system leading to measurable increases in cortical motor activity [113]. In this technique, the effect of carbonated water on pharyngeal mucosa acts in a similar but less controlled manner to electrical stimulation in PES. However, PES is thought to be safer than carbonated water because it does not carry the associated risk of accidental aspiration during a course of therapy in patients with neurogenic dysphagia [113].

Other peripherally acting neuromodulation techniques exist such as transcutaneous electrical stimulation (TCS) [114]. However, these tend to be more indirect in the application of electrical energy. In TCS, electrical energy is applied to the skin with the aim of modulating sensorimotor activity in underlying swallowing associated structures [114]. At present, more evidence exists in support of the efficacy of PES than TCS.

### Healthy Participant Studies

The first human PES report was a 1998 study by Hamdy et al. [102]. In this study, PES was administered for 10 min at a frequency of 10 Hz to eight healthy participants. Motor cortical excitation was assessed using single-pulse TMS targeted at pharyngeal motor cortical areas to measure pharyngeal MEP (PMEP) amplitudes. PMEP recordings were taken at 0-, 30- and 60 min post-stimulation [102]. It was found that PES resulted in a significant increase in PMEP amplitudes at 30 min but had returned to baseline by 60 min. Interestingly, no changes were observed in PMEP latency or latency of the Trigeminal (Cranial nerve V) and Vagus (Cranial nerve X) nerves post-PES [102]. This indicated the increases in PMEP amplitude observed were primarily caused by cortical excitation as opposed to any brainstem effects. Subsequently, in 2002 Fraser et al., sought to further refine PES stimulation parameters [103]. In their study, PES was administered for 5, 10 and 20 min to eight healthy participants at frequencies of 1 Hz, 5 Hz, 10 Hz, 20 Hz and 40 Hz at intensities of 25%, 50% and 75% of maximum tolerated intensity. PES at a frequency of 5 Hz administered for 10 min at an intensity of 75% was found to provoke the largest increases in PMEP amplitude. Significant increases in PMEP amplitudes were observed to persist at 60 min post-stimulation [103]. In contrast to the earlier study by Hamdy et al., no significant increase in excitation was found to occur at a frequency of 10 Hz. In 2003, Fraser et al., compared the cortical excitatory effects of PES to water swallowing in a group of eight healthy participants [104]. They found that while both PES and water swallowing were able to increase PMEP amplitude, the pattern of excitation differed depending on the approach. PES provoked a significant increase in PMEP amplitude at 60 min, a delayed cortical response, while water swallowing prompted a significant immediate increase in excitation which dissipated by 15 min [104]. A similar study was conducted in 2016 by Magara et al. [113]. In this study, they studied the cortical response of 14 healthy participants to PES, still water or carbonated water. They found PES, when applied alone, provoked long-term cortical excitation, but combining PES with carbonate water or still water did not, suggesting that PES may be most effective when used as a standalone treatment [113].

Prior to the use of PES in patients with neurogenic dysphagia, the ability of PES to reverse the suppressive effects of a cortical “virtual lesion” was studied [115]. This work was done in a group of 13 participants in 2010 [115]. PES was found to result in a significant restoration in PMEP amplitude and swallowing behaviour (both normally disrupted by the “virtual lesion”), as assessed using a swallowing accuracy task, compared to sham [115].

### Neurogenic Dysphagia Studies

#### Stroke Studies

Several studies have been performed using PES to improve swallowing function in patients with PSD [103, 106, 115, 116]. The first study of this type was performed in 2002 by Fraser et al. [103]. In this study, 16 patients with PSD were divided into two unequal groups. Six patients received sham PES where the catheter was inserted but the signal generator was not switched on. Ten patients received active PES given as per the optimal parameters determined earlier from the initial group of 8 healthy participants. Measurements of PMEP amplitude were taken at baseline, immediately post-stimulation and at 60 min post-stimulation, while VFS recordings were taken at baseline and 60 min post-stimulation [103]. No significant change in PMEP amplitudes was seen between the sham and active stimulation groups but a significant improvement in Penetration Aspiration Scale (PAS) [117] scores was observed in the active group that critically correlated with increased cortical excitability within both hemispheres [103]. Subsequently, in 2010, Jayasekeran et al. performed a larger PSD study in a group of 50 patients [115]. This study, split into two parts, aimed to determine the optimal treatment regime for PES (*n* = 22) and the long-term effects of PES on swallowing function (*n* = 28) [115]. The dose response part of the study found that a regime of one 10-min session of PES per day for 3 days gave the greatest reduction in PAS [115]. Details as to the five stimulation parameters studied can be found in Table 3. In the second half of the study, the optimal PES treatment regime was compared against sham in a group of patients with VFS being performed at baseline and 2 weeks post-PES [115]. Active PES resulted in a statistically significant decrease in PAS scores [115]. In 2016, Vasant et al., performed a similar RCT which aimed to investigate a longer time period of effect of PES on swallowing function with functional score measurements and VFS recordings being taken at baseline, 2 weeks and 3 months post-stimulation [116]. In contrast to earlier studies, despite positive trends, no significant difference was seen between active PES and sham for PAS scores or for dysphagia severity rating scale (DSR) values. In the same year, Bath et al., published a larger RCT incorporating 162 patients with PSD recruited from multiple international stroke units [106]. This study proved to be neutral with no difference being seen to emerge between patients given active PES and sham [106].

**Table 3:** Summarized findings on the effects of PES for neurogenic dysphagia

	PES intensity and duration	Number of sessions	Sample size	Demographics	Outcome measures	Post disease onset/duration	Major outcomes
*Healthy participant studies*							
**Hamdy et al. 1998 **[102]	**10 Hz** Set intensity at the limit of participant perception 10 min	1	8	8 males Mean age 33	PMEP amplitude Measurements at 0 min and 30 min post-stimulation	–	Significant increase in PMEP amplitudes immediately after PES and at 30 min
**Fraser et al. 2002** [103]	**Part 1** Each participant received 1, 5, 10, 20, 40 Hz Each participant received 5, 10 and 20 min of PES Each participant received 25, 50 and 75% of maximally tolerated PES intensity **Part 2** **5 Hz** At 75% maximal tolerated intensity 10 min	**Part 1** **(*PES dose response study*)** 11 sessions of active PES **Part 2** **(*fMRI study*)** 1 session of active PES and 1 session of sham PES prior to having a brain fMRI	**Part 1** 8 **Part 2** 8	**Part 1** 2 females 6 males Mean age 26 **Part 2** 1 female 7 males Mean age 26	**Part 1** PMEP amplitude Measurements at 0 min and 30 min post-stimulation **Part 2** BOLD signal intensity	**Part 1** – **Part 2** –	**Part 1** 5 Hz PES at 75% of maximally tolerated intensity for 10 min led to the greatest increase in PMEP amplitudes **Part 2** Active PES was associated with a bilateral increase in BOLD signal intensity over sensorimotor areas
**Fraser et al. 2003** [104]	**5 Hz** At 75% maximally tolerated intensity 10 min	3 1 = water swallowing 1 = PES 1 = water swallowing post pharyngeal anaesthesia	8	2 females 6 males Mean age 31	PMEP amplitude Measurements at 0, 15, 30, 45 and 60 min	–	Significant increases PMEP amplitudes post-PES and water swallows. However, no similar changes following pharyngeal anaesthesia
**Jayasekeran et al. 2010** [115]	**Parts 1** **5 Hz** At 75% maximally tolerated intensity 10 min	**Part 1** **(*PES virtual lesion study*)** 2 1 = active PES post virtual lesion, 1 = sham	**Part 1** 13	**Part 1** 6 females 5 males	**Part 1** PMEP amplitude Swallowing reaction timed task Measurements at 10-min intervals	**Part 1** **–**	**Part 1** Active PES reversed the suppressive PMEP and disruptive behavioral effects of a cortical virtual lesion
**Magara et al. 2016** [113]	**5 Hz** At 75% maximally tolerated intensity 10 min	4 1 = PES 1 = sham + carbonated water 1 = PES + carbonated water 1 = PES + normal water	14	3 females 11 males Mean age 27	PMEP amplitude Measurements at 0, 30 and 60 min	–	PES (alone) produced significant increases in cortical PMEP amplitude. However, this was not the case for PES in combination with other approaches
*Post-stroke dysphagia studies*							
**Fraser et al. 2002** [103]	**Part 3** Intensity delivered as per part 2	**Part 3** 1 2 groups, active PES and sham	**Part 3** 16	**Part 3** 6 females 10 males Mean age 74	**Part 3** PMEP amplitude VFS PAS and swallowing timing Measurements at 0 min and 1-h post-stimulation	**Part 3** No timing specified Mean: 4 days post event	**Part 3** Active PES resulted in a significant improvement in PAS and swallow timing measures
**Jayasekeran et al. 2010** [115]	**Part 2** As per part 1 at varying doses **Part 3** **5 Hz** 1 × per day for 3 days At 75% maximal tolerated intensity 10 min	**Part 2** **(*PES dose response study*)** 0, 3, 5, 9 or 15 (total sessions) participants sorted into five groups 0 (controls), 1 × per day for 3 days 1 × per day for 5 days 3 × per day for 3 days 3 × per day for 5 days ***Part 3*** **(*PES patient study*)** 1 randomized to active or sham	**Part 2** 22 **Part 3** 28 (randomized to active or sham)	**Part 2** 15 males 7 females Mean age 73 **Part 3** Mean age 75 active group 74 sham group	**Part 2** VFS PAS **Part 3** VFS PAS	**Parts 2 and 3** No timing specified	**Part 2** A PES regime of 1 × per day for 3 days is effective in improving PAS and its effects are no worse than approaches with more prolonged stimulation **Part 3** Active PES resulted in a significant improvement in PAS and reduced length of hospital stays
**Vasant et al. 2016** [116]	**5 Hz** 1 × per day for 3 days At 75% maximal tolerated intensity 10 min	3 2 groups, active and sham	36	14 females 22 males Mean age 71	DSR VFS PAS Measurements at 2 weeks and 3 months post-PES	≤ 6 weeks post event	Non-significant findings. However, there was a trend towards PAS and DSR improvement with active stimulation
**Bath et al. 2016** [106]	**5 Hz** 1 × per day for 3 days At 75% maximal tolerated intensity 10 min	3 2 groups, active and sham	162	68 females 94 males Mean age 74	VFS PAS Measurements at 2 weeks and 3 months	≤ 42 days post event	No significant differences in treatment effects between active and sham groups
*Other neurogenic dysphagia studies*							
**Restivo et al. 2013** [120]	**5 Hz** 1 × per day for 5 days At 75% maximal tolerated intensity 10 min	5 (10 patients received active PES and 10 received sham)	20	Patients with Multiple Sclerosis (MS) and dysphagia 13 females 7 males Mean age 39.7 14 patients with relapsing remitting MS and 6 with secondary progressive	VFS PAS EMG recordings of laryngeal excursion, submental muscles and cricopharyngeus	Mean MS duration 9.8 years Mean dysphagia duration 22 months	Active PES lead to significant improvement in PAS scores compared to baseline at 5 days, 2 weeks and 4 weeks Active PES lead to significant improvement in EMG parameters at 5 days, 2 weeks and 4 weeks compared to baseline
**Sasegbon et al. 2021** [58]	**5 Hz** At 75% maximal tolerated intensity 10 min	1 (3 patients received active PES and sham in a cross-over manner)	3	Patients with Parkinson’s disease and dysphagia 3 males Mean age 75	VFS PAS VFS swallow timing measurements at baseline, 0 and 30 min PMEP amplitude at times detailed above	Mean Parkinson’s duration 2 years	No statistical testing applied due to the small sample size. Small visible trend towards improvement in PAS for thin fluids and paste Otherwise PES well tolerated

Significance was set at 0.05

*DSR* dysphagia severity rating scale, *EMG* electromyography, *MS* multiple sclerosis, *PAS* penetration aspiration score, *PES* pharyngeal electrical stimulation, *PMEP* pharyngeal motor evoked potential, *VFS* videofluoroscopy

In 2017, Muhle et al., published a study investigating the effects of PES on salivary substance P levels in 23 patients with PSD [112]. In the study PES resulted in a significant increase in salivary substance P levels [112]. With regards to functional imaging studies, studies in patients with PSD have confirmed that PES induced increases in PMEP amplitude, first described in healthy participant studies, occur alongside increases in motor cortical blood flow [103].

Although several meta-analyses have been performed to evaluate the clinical efficacy of dysphagia treatments, few have incorporated PES [35, 36]. Furthermore, only the meta-analysis performed by Cheng et al., in 2021 looked at PSD in isolation [35]. In contrast, Speyer et al. combined outcomes from all causes of neurogenic dysphagia [36, 56]. Cheng et al. [35] showed PES exerts a significant beneficial effect at improving PSD. When all current studies are considered as a whole, the role of PES in the treatment of PSD remains broadly positive. It should be noted that stroke patients with more severe dysphagia leading to tracheotomy appear to show most benefit. The currently ongoing Pharyngeal Electrical stimulation for Acute Stroke dysphagia Trial (PhEAST; ClinicalTrials.gov Identifier: NCT05190718) study, which is a large-scale multi-centre study involving 50 sites over 4 countries (UK, Austria, Denmark, Germany) with a target sample size of 800 patients with post-stroke dysphagia, should hopefully cast some light on this area. Details as regards the studies performed utilising PES as a treatment for PSD can be seen in Table 3.

#### PES to Increase Airway Safety in Tracheostomized/Intubated Patients

In patients with tracheostomies and dysphagia, PES administered as per the regime developed by Fraser et al., in 2002 [103] and further refined by Jayasekeran et al., in 2010 [115], has been shown to be beneficial in aiding extubation [105, 118]. In more detail, Suntrup et al., [118] and Dziewas et al. [105], randomized 30 and 69 patients with neurogenic dysphagia and tracheostomies to either PES or sham stimulation. In both studies, PES, compared to sham, resulted in a significant increase in extubation [105, 118]. A subsequent pilot study by Koestenberger et al., in 15 patients compared against a historic control group, PES was found to reduce rates of re-intubation and post-extubation pneumonia compared to sham [119].

#### Other Disease Processes

In 2013, Restivo et al., used PES to treat dysphagia in 20 patients with Multiple Sclerosis (MS) [120]. In this sham-controlled study, PES resulted in significant improvements in electromyographic (EMG) recordings from external pharyngeal musculature and PAS scores at 2 weeks and 4 weeks post administration compared to sham [120]. More recently, a pilot study was published by Sasegbon et al., in 2021 in which PES was used to treat patients with Parkinson’s associated dysphagia [121]. Although the technique was well tolerated in this patient group, low recruitment numbers resulted in a limited interpretation of the effects of PES in this population [121].

## Paired Associative Stimulation

Paired associative stimulation is another form of neuromodulation that combines peripheral stimulation of target muscles with central stimulation of the cortical regions that represent the target muscle. Singh et al., first discovered that paired associative stimulation can induce neuroplasticity changes in the human swallowing system [122]. They found that an inter-stimulus interval of 100 ms was optimal for inducing bilateral enhancement in pharyngeal cortical excitability and such effect lasted for at least 2 h. Importantly, this study provided evidence that paired associative stimulation resulted in a focal reduction in brain glutamate as detected by magnetic resonance spectroscopy (MRS), suggesting that paired associative stimulation-induced changes were mediated by glutamate and may involve long-term potentiation- (LTP−) like synaptic strengthening in the pharyngeal motor cortex. Another study by Michou et al., found that 10 min of paired associative stimulation delivered to the dominant swallowing (pharyngeal) hemisphere increased the pharyngeal cortical excitability and improved the number of on-target swallows in a swallowing task in healthy individuals [123]. Furthermore, their findings showed that paired associative stimulation can reverse the neurological and behavioural disruptions induced by “virtual lesion”.

In patients with chronic PSD, paired associative stimulation applied over the unaffected hemisphere enhanced the cortical excitability of both hemispheres, and such changes were accompanied with reduced aspiration and penetration and improved swallowing timings. In a further randomized controlled cross-over study, Michou et al., found that paired associative stimulation and PES showed comparable effects in increasing cortical excitability and improving swallowing safety in patients with chronic PSD [40]. However, the evidence of paired associative stimulation as a dysphagia treatment is relatively weak compared to other forms of neuromodulation because of the small sample sizes and limited number of RCTs. Further large-scale clinical trials are warranted to evaluate its clinical value for dysphagia.

## Limitations of Neuromodulation Techniques and Implications

Despite the growing body of research in recent years and the seemingly promising findings of NIBS treatments for dysphagia, a few issues remain unresolved to date which calls for further investigation. Firstly, there is no consensus on the optimal rTMS or tDCS protocols for facilitating functional recovery in patients with neurogenic dysphagia. The lack of consensus may be attributed to the heterogeneity in stimulation protocols, patient characteristics and outcome measures, which makes direct comparisons of findings across studies challenging. The issue of methodological diversity is particularly problematic for studies with tDCS. As mentioned earlier, the position and size of electrodes are of paramount importance when studying the neuromodulation effects. However, most tDCS studies on the human swallowing system do not report details on electrode montages. Therefore, questions regarding how the electric fields and current flows are affected by the electrode placement, and the neuromodulation effects on regions between the electrodes remain unanswered. Furthermore, there is limited work around the longer term (beyond 3 months) effects of these treatments. It is therefore unclear whether additional dosage may be essential to maintain treatment benefit in the long run.

Apart from study designs, individual variability in responsiveness to NIBS treatments may also play a role in the mixed findings [124–127]. Studies have suggested that the responsiveness to NIBS treatments are dependent on several intrinsic and extrinsic factors, including age, attention, gender, pharmacological influences, genetics, time of day, neurological recruitment during rTMS, and proximity of brain lesion to the stimulation target [128–130]. Of relevance to swallowing, a few studies have explored the contributing factors to the variability in responsiveness to NIBS treatments [88, 127]. Raginis-Zborowska et al., found that only 13% of participants showed expected bidirectional responses to both 1 Hz and 5 Hz rTMS applied over the pharyngeal motor cortex [127]. The responses to these protocols were determined in part by single nucleotide polymorphisms (SNPs) in the genes Catechol-O-Methyltransferase (*COMT*) and Dopamine Receptor D2 (*DRD2*). Another study by Hwang et al., also suggested that *COMT* Val158Met polymorphism may affect the outcomes of tDCS. They found that individuals with *Val/Val* polymorphism showed improvements in cortical excitability and swallowing function following anodal tDCS but those with *Met* allele did not [88]. These findings suggested that genetic predispositions may influence the NIBS treatment outcomes and certain genes may act as biomarkers for responsiveness to these treatments.

Another factor that may affect the treatment outcomes is the neuronal state preceding NIBS. Cheng et al., discovered that when the pharyngeal motor cortex is preconditioned with high or low-frequency rTMS, the response towards a subsequent rTMS can be potentiated compared to no preconditioning [131]. This finding provided not only a potential explanation for the response variability, but also a novel strategy to minimize it and maximize beneficial outcomes of NIBS treatments. Future studies should explore the possibility of adopting an individualized treatment approach that caters for individual differences in genetic predispositions and neuropathophysiology.

## Practical Considerations for Using Neuromodulation in Clinical Practice

Although recent research has suggested promising therapeutic potential of PES, rTMS and tDCS for neurogenic dysphagia, there are several practical concerns that needs to be considered when adopting these techniques in clinical practice. Firstly, some of these techniques have not been approved as a treatment for dysphagia by regulatory bodies such as the European Commission in Europe or the Food and Drug Administration (FDA) in the United States. PES has received Conformitè Europëenne (CE) mark approval as a dysphagia treatment, but it has not yet been approved by the FDA. RTMS is a CE and FDA-approved treatment for major depressive disorder, but relevant approval has not been granted for rTMS as a dysphagia treatment. Similarly, tDCS is a CE-approved treatment for major depressive disorders in the UK, EU, Australia and Mexico, but it has not been approved for dysphagia, nor has it been approved by the FDA.

Another practical concern is that these treatments can be expensive. The costs of hardware, which range from USD 1500 (tDCS) to USD 35,000 (rTMS), and the running costs associated with training of personnel who delivers the treatments and maintenance of the devices can be high, making it challenging for clinicians to adopt these treatments in clinical practice. Moreover, since these treatments are not approved by the FDA, most insurance companies in the United States do not cover the medical costs, and as such, patients may be more reluctant to seek these treatments. Nonetheless, if future studies provide sufficient evidence that these treatments can help patients maintain safe swallowing in the longer term, with reduced risk of hospital admission due to dysphagia-related complications, they may improve the cost-effectiveness of dysphagia care for patients and the society in the long run. Table 4 summarizes the similarities and differences of the three neuromodulation techniques discussed in this review.

**Table 4:** Comparisons among repetitive transcranial magnetic stimulation (rTMS), transcranial direct current stimulation (tDCS) and pharyngeal electrical stimulation (PES)

	Repetitive transcranial magnetic stimulation (rTMS)	Transcranial direct current stimulation (tDCS)	Pharyngeal electrical stimulation (PES)
Mode of neuromodulation	Central neuromodulation	Central neuromodulation	Central and peripheral neuromodulation
Proposed mechanisms of action	Electric current is induced in the brain tissue through electromagnetic induction Strong electric current depolarizes nerve cells and triggers action potentials	Electric current applied indirectly onto the brain through scalp surface electrodes Weak electric current influences membrane polarization subthreshold	Electrical stimulation of pharyngeal mucosa 5 Hertz found to provoke cortical excitation
	Can induce neuroplastic changes in the human swallowing system	Can induce neuroplastic changes in the human swallowing system	Can induce neuroplastic changes in the human swallowing system
Equipment	TMS stimulator containing a capacitor discharge system External coil of wire	Stimulator delivering direct electric current Surface positive (anode) and negative (cathode) electrodes	A disposable catheter containing ring electrodes attached to an electrical generator
Regulatory body approval	CE and FDA-approved treatment for major depressive disorder No CE or FDA approval for dysphagia	CE-approved treatment for major depressive disorder No CE or FDA approval for dysphagia	CE-approved treatment for dysphagia No FDA approval for dysphagia
Cost	Approximately 35,000 USD	Approximately 1500 USD	Disposable catheters: Yet to be determined Electrical signal generator: Yet to be determined

*CE* Conformitè Europëenne, *FDA* Food and Drug Administration

Several published guidelines on the use of neuromodulation for dysphagia may be helpful in guiding the decision-making process in clinical practice. The German Society of Neurology recommended that (1) before initiating dysphagia treatment with a neurostimulation approach, the pattern of swallowing impairment should be determined as precisely as possible; (2) all neurostimulation methods should be used as a supplement to the behavioral swallowing therapy; (3) due to limited data, neurostimulation methods in principle should be used in clinical trials or registries; and (4) pharyngeal electrical stimulation (PES) should be used to treat dysphagia in tracheotomized stroke patients with supratentorial lesion. Participation in prospective clinical registries is recommended” ([132], p. 20).

Similar recommendations have also been outlined in the latest European Stroke Organisation and European Society for Swallowing Disorders guideline for the diagnosis and treatment of post-stroke dysphagia [133]. This guideline recommended that (1) treatment with neuromodulation techniques for patients with PSD should be conducted within a clinical trial setting; (2) rTMS, tES, tDCS and PES can be used as adjunction to conventional dysphagia treatments; and (3) PES is recommended to accelerate decannulation for tracheotomized stroke patients with severe dysphagia (p. CII–CIII).

## Conclusions

Neuromodulation techniques have become a hot topic in the field of neurogenic dysphagia rehabilitation in recent years. In this review, we have discussed the potential mechanisms of three major neuromodulation techniques and their effects on the human swallowing motor system. PES is a peripherally delivered technique whereas tDCS and rTMS are delivered directly to the brain. Common to these techniques is the ability to manipulate or alter the swallowing neuromotor system, as evidence in the changes in cortical excitability, swallowing-related biochemistry and swallowing behaviour following stimulation that last longer than the duration of stimulation. These long-term changes are of paramount importance as a therapeutic tool for dysphagia because they imply that neuroplasticity mechanisms, which lead to sustained functional recovery, may have been facilitated by these techniques. Indeed, the clinical evidence with patients with neurogenic dysphagia suggests that these techniques have modest but significant benefits in improving swallowing function. Despite this, there are a few limitations, including the heterogeneity in study designs and variability of responsiveness, that remain unresolved, which call for further investigation. Future research and clinical directions should focus on adopting an individualized approach to these techniques that take into consideration the differences in genetic predispositions and neuropathophysiology among patients. Finally, practical concerns regarding regulatory body clearance and treatment costs must be considered when using neuromodulation treatments in clinical practice.
